# Linear and nonlinear multidimensional functional connectivity methods reveal similar networks for semantic processing in EEG/MEG data

**DOI:** 10.3389/fnhum.2025.1533034

**Published:** 2025-07-30

**Authors:** Setareh Rahimi, Rebecca L. Jackson, Olaf Hauk

**Affiliations:** ^1^MRC Cognition and Brain Sciences Unit, University of Cambridge, Cambridge, United Kingdom; ^2^Department of Psychology and York Biomedical Research Institute, University of York, York, United Kingdom

**Keywords:** nonlinear, event-related connectivity, functional connectivity, semantic representation, semantic control, multidimensional, EEG, MEG

## Abstract

**Introduction:**

Investigating task- and stimulus-dependent connectivity is key to understanding how the interactions between brain regions underpin complex cognitive processes. Yet, the connections identified depend on the assumptions of the connectivity method. To date, methods designed for time-resolved electroencephalography/magnetoencephalography (EEG/MEG) data typically reduce signals in regions to one time course per region. This may fail to identify critical relationships between activation patterns across regions. Time-Lagged Multidimensional Pattern Connectivity (TL-MDPC) is a promising new EEG/MEG functional connectivity method improving previous approaches by assessing multidimensional relationships between patterns of brain activity. However, TL-MDPC remains linear and may therefore miss nonlinear interactions among brain areas.

**Methods:**

Thus, we introduce Nonlinear TL-MDPC (nTL-MDPC), a novel bivariate functional connectivity method for event-related EEG/MEG applications, and compare its performance to the original linear TL-MDPC. nTL-MDPC describes how well patterns in ROI X at a time point $t_{x}$ can predict patterns of ROI Y at a time point $t_{y}$ using artificial neural networks.

**Results:**

Applying this method and its linear counterpart to simulated data demonstrates that both can identify nonlinear dependencies, with nTL-MDPC achieving up to ~0.75 explained variance under optimal conditions (e.g., high SNR), compared to ~0.65 with TL-MDPC. However, with a sufficient number of trials- e.g., a trials-to-vertex ratio ≥10:1 - nTL-MDPC achieves up to 15% higher explained variance than the linear method. Nevertheless, application to a real EEG/MEG dataset demonstrated only subtle increases in nonlinear connectivity strength at longer time lags with no significant differences between the two approaches.

**Discussion:**

Overall, this suggests that linear multidimensional methods may be a reasonable practical choice to approximate brain connectivity, given the additional computational demands of nonlinear methods.

## 1 Introduction

Cognitive processes originate from dynamic interactions among multiple brain regions (Passingham et al., 2002; Bullmore and Sporns, 2009). Functional connectivity methods are key to understanding how brain regions interplay to generate these processes. The most common functional connectivity methods are unidimensional, summarising information in a brain area by collapsing the pattern of activity across all the voxels or vertices within a region. However, recently ‘multidimensional’ methods have been proposed, avoiding the loss of valuable information in the brain response patterns by making use of multiple time courses per region (Anzellotti and Coutanche, 2018; Basti et al., 2020; Rahimi et al., 2023). These methods include Multivariate Pattern Dependence (MVPD; Anzellotti et al., 2017a) for fMRI and Time-Lagged Multidimensional Pattern Connectivity (TL-MDPC) for time-resolved methods, such as EEG and MEG (Rahimi et al., 2023). In both cases, these multidimensional connectivity methods successfully identified richer connectivity than unidimensional methods.

Despite this success, it remains possible that nonlinear connections are being missed when utilising linear methods to assess the relationship between these brain response patterns. While linear approaches are computationally efficient and their resulting transformation matrices are easily interpretable, they may miss important nonlinear relationships. This is critical as neuronal activation is nonlinear, with a neuron’s firing rate depending on the nonlinearly transformed sum of the inputs to the neuron (McCulloch and Pitts, 1943; Hebb, 2005). While EEG/MEG data is not focused on this cellular level, broader systems-level operations, such as regional spike counts, result from these cellular nonlinearities. Moreover, nonlinear transformations are considered necessary for the kinds of computations the human brain performs, such as the combination of sensory features into a multimodal conceptual structure in the case of semantic processing (Lambon Ralph et al., 2010; Hauk et al., 2023). Indeed, animal studies have demonstrated the importance of nonlinear relationships in the visual ventral stream (DiCarlo et al., 2012). For this reason, a nonlinear version of MVPD was introduced for fMRI connectivity assessment (Anzellotti et al., 2017b). To do so, a nonlinear artificial neural network (ANN) replaced linear regression as the method to characterise statistical relationships between the multidimensional activity patterns of two ROIs. When applied to resting state fMRI data, the nonlinear connectivity method identified more significant pattern dependencies than its linear counterpart. While this demonstrates the importance of assessing nonlinear relationships between regional activity patterns, there are currently no comparable methods for time-resolved EEG/MEG data.

Here, we address this need by developing a nonlinear variant of TL-MDPC and testing whether it can capture connectivity relationships that linear methods may miss. To do so, we build upon the TL-MDPC method recently developed by Rahimi et al. (2023) and applied to event-related EEG/MEG data in source space. TL-MDPC estimates the transformation between patterns for pairs of brain regions and pairs of time lags, utilising the cross-validated explained variance (EV) of these transformations as a metric for connectivity strength. The result is a temporal transformation matrix (TTM), a plot of the connectivity strength between ROI X and Y at each possible combination of time points. Unlike other multidimensional EEG/MEG connectivity approaches (Pascual-Marqui, 2007; Ewald et al., 2012; Basti et al., 2018), TL-MDPC does not have to be computed for narrow frequency bands and maintains the temporal resolution needed to estimate the time course of connectivity. Following Anzellotti et al. (2017b), we extended the linear approach of Rahimi et al. (2023) to create nonlinear Time-Lagged Multidimensional Pattern Connectivity (nTL-MDPC) by investigating the relationships between regions using an ANN, as opposed to the cross-validated ridge regression used previously. Thus, nTL-MDPC estimates how well patterns in ROI X at time point $t_{x}$ can predict patterns in ROI Y at time point $t_{y}$ through a nonlinear mapping. This should allow the identification of both linear and nonlinear relationships between multidimensional patterns of activity in pairs of brain regions and across latencies. Like TL-MDPC, nTL-MDPC is a bivariate functional connectivity method suitable for event-related datasets. In contrast to similar methods previously applied to fMRI data (Anzellotti et al., 2017a, 2017b; Basti et al., 2019), it characterises pattern connectivity over time, i.e., for different time lags, as well as over space. nTL-MDPC can estimate the full vertex-to-vertex transformations between ROIs. However, EEG/MEG source estimates have inherently limited spatial resolution, which depends on many factors, including source location, orientation, and signal-to-noise ratio (Molins et al., 2008; Hauk et al., 2019; Samuelsson et al., 2021). Consequently, source signals in different voxels within an ROI carry redundant information (“leakage”). To reduce redundancy and computational cost arising from spatial leakage and smoothness in EEG/MEG source estimates, we followed the same approach utilised in TL-MDPC (Rahimi et al., 2023), selecting the most informative vertices within each ROI via unsupervised k-means clustering. This was done by clustering vertices based on their activation profiles across trials and retaining, from each cluster, the vertex with the highest trial-wise variance—likely to contribute most to connectivity estimates. This approach performs *feature selection*, preserving the vertex-wise spatial structure without transforming the data into a new space, as would be the case with *feature extraction* techniques such as PCA or SVD (Khalid et al., 2014). We selected k-means for its simplicity, computational efficiency, and fast convergence, making it particularly suited to high-dimensional EEG/MEG source data. The number of clusters (typically 5–13) was determined using the Elbow method, chosen for its simplicity, reproducibility, and alignment with our prior work. We then estimate the nonlinear mappings between the sub-sampled patterns using a cross-validated ANN regression method, employing 10-fold cross-validation and regularisation to avoid overfitting.

We tested the performance of nTL-MDPC by comparison to its linear counterpart in both simulations and a real EEG/MEG dataset. The simulations allowed us to demonstrate that nTL-MDPC was able to capture both linear and nonlinear relationships between the pattern of responses in two signals, while avoiding false positives. Moreover, we tested whether nTL-MDPC indeed detects the nonlinear relationships significantly better than TL-MDPC. In our real EEG/MEG data analysis, we assessed whether this hypothesised improvement in the ability to detect nonlinear relationships with nTL-MDPC would result in the identification of additional task-related functional connectivity differences. We contrasted dynamic network connectivity between a more and a less semantically demanding word-based decision task with nTL-MDPC, as we previously did with TL-MDPC and directly compared the results (Rahimi et al., 2023). This comparison provides an ideal test case as both tasks require a nonlinear transformation between visual input and semantic knowledge, yet the demands on the semantic network are greater in one condition. We hypothesised that nTL-MDPC would capture nonlinear multidimensional relationships within the brain that its linear counterpart is insensitive to.

## 2 Materials and methods

We investigated whether nTL-MDPC detects more connectivity than the linear TL-MDPC method in both simulated data and a real EEG/MEG dataset. We first introduce the rationale and procedures that are shared between both methods. In both cases, we first need to (1) prepare the patterns at each time in each region, (2) attempt to find a mapping/transformation between two patterns at two time points, (3) predict the target ROI’s output, (4) and finally measure the explained variance (EV) between the real and predicted output, as the connectivity metric.

**Figure 1:**
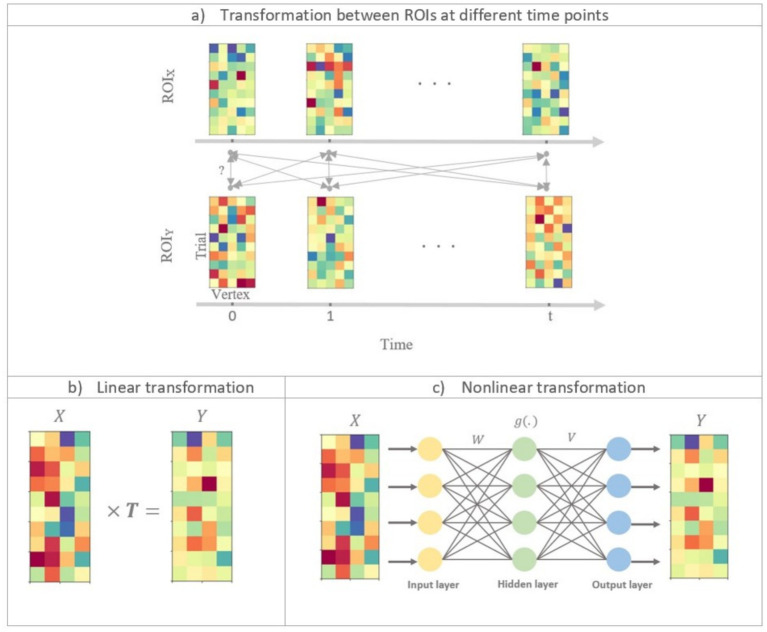
Illustration of linear and nonlinear time-lagged multidimensional pattern connectivity approaches. **(a)** The principle of TL-MDPC is displayed. We assess the relationship between activity patterns in ROI X and ROI Y at different time lags. Each matrix indicates activity patterns in one ROI at one time point, with rows in each matrix indicating activation across different trials, and columns representing activation over different vertices in the ROI. Bidirectional arrows represent possible transformations and dependencies between patterns. **(b)** Illustration of using TL-MDPC to detect the linear transformations T between patterns using ridge regression (as in Rahimi et al., 2023). **(c)** Illustration of the novel nTL-MDPC method to detect nonlinear (and linear) transformations between patterns using an artificial neural network.

Both methods have the same aim. Let us consider that X and Y are activity pattern matrices of ROI X and ROI Y at the time points $t_{x}$ and $t_{y}$ of size $\;n_{t}\times n_{X}$ and $n_{t}\times n_{Y}$ , respectively, where $n_{t}$ is the number of trials, and $n_{X}$ and $n_{Y}$ are the number of vertices in the two regions. Figure 1a represents activity patterns in ROI X and ROI Y across time. We intend to find out whether there is an all-to-all mapping between the patterns of responses in the two ROIs at different latencies. Simply put, we assess how well the patterns in ROI Y at the time point $t_{y}$ can be predicted from the patterns in ROI X at the time point $t_{x}$ , and the other way around, through a transformation (in each direction).

To do so, we first need to address the spatial resolution of EEG/MEG. These signals are inherently smooth and have limited spatial resolution (Palva et al., 2018; Hauk et al., 2019). As a result, not all vertices are independent. To deal with this issue, unsupervised k-means clustering is employed as a “feature selection” approach to sub-sample the most informative vertices within each ROI (Rahimi et al., 2023). Using this approach, vertices serve as samples/observations, and trials serve as features. Thus, we group all vertices into k clusters, so that all vertices with similar activation profiles across trials are within one cluster. To find out the optimum number of clusters, the elbow method (Ng, 2012) was used. There are several ways one could pick a representative vertex within each cluster, including computing the mean of all vertices, the centroid of the cluster, or the vertex with the highest variance. Following Rahimi et al. (2023), we pick the vertex with the highest variance as this allows us to keep the patterns in the genuine pattern space while estimating the transformations between regions. As a result of clustering, the patterns X and Y at time points $\;t_{x}$ and $t_{y}$ are now of size $n_{t}\times n_{x}$ and $n_{t}\times n_{y}$ , where $n_{x}$ and $n_{y}$ are the number of clusters in ROI X and Y . Notably, the clustering is performed separately for each time point.

We are then able to estimate the transformations between regions. Using the dimensionality-reduced patterns in ROI X and Y at two specific time points ( X and Y ), we can extract the transformation matrix T , allowing us to predict the patterns in ROI Y ( ${\hat{Y}}_{\text{test}}$ ) through *T* and the test subset $X_{\text{test}}$ ( $X_{\text{test}}$ is obtained from X by selecting a subset of rows). We can then measure the explained variance (EV) between the real output, $\;Y_{\text{test}}$ , and the predicted output, and use this as a measure of connectivity. The highest score would be 1, suggesting a very strong relationship (either linear or nonlinear) between patterns, while close to zero values reflect very weak (or no) connectivity. In the following, we replace negative values of EV by zero since they indicate that the data could not fit the model at all, and a variation in negative EVs is not meaningful. Additionally, since our measurements are not directional, but a reflection of statistical dependencies between each pair, we predicted X from Y and Y from X , and reported the average of the resulting EVs.

For each pair of ROIs, we repeat this procedure for all possible pairs of time points. The resulting EVs constitute the Temporal Transformation Matrix (TTM) for ROI pair X and Y. Thus, different areas in this matrix reflect connectivity at different time lags; the diagonal entries of TTMs show simultaneous connectivity between ROI X and Y patterns, the upper diagonal represents connectivity where Y is ahead, and lower diagonal shows connectivity where X is ahead.

### 2.1 The nTL-MDPC method

The nTL-MDPC method differs from TL-MDPC in one critical aspect, namely the method used to estimate the transformations. While the linear TL-MDPC uses cross-validated ridge regression (Hoerl and Kennard, 1970) (Figure 1b), the nonlinear method employs ANNs to extract the mapping between X and Y (Figure 1c). ANNs can estimate the relationship between linear and nonlinear multidimensional time courses (Singh et al., 2003). By gradually changing their connection weights to reduce error in their output, ANNs learn to map from a given input to a target output, in this case from the multidimensional activity patterns in one ROI at one time point to those in another ROI at one time point. As shown in Figure 1c, the activation in each unit in the ANN (other than input units) is generated through the weighted sum of the activity of connected units in the prior layer. In the hidden layer (i.e., the units which do not directly receive input or give output), unit activity is further determined with an additional step whereby every node passes the weighted sum of the inputs to that unit through a nonlinear function. In cases where the unknown coefficients outnumber the observation samples (an ill-posed or underdetermined problem), a regularisation procedure is needed. For this purpose, we used a cross-validated regularised ANN to avoid overfitting. As in the linear method, the explained variance of the transformations will serve as the connectivity metric.

#### 2.1.1 Organisation of the ANN

Figure 1c illustrates the general framework of a feedforward ANN (Venkatesan and Anitha, 2006) with one hidden layer. We used a nonlinear regression implemented in Python^1^ using ANN. We utilised a tangent hyperbolic activation function as this is widely used (Sharma et al., 2017). The number of units in the hidden layer was set to be the average of the number of nodes in the input and output layers. The network is trained using a standard back-propagation learning algorithm (Rojas, 1996). The solver for weight optimisation was set to “LBFGS” (Limited-memory Broyden–Fletcher–Goldfarb–Shanno), an unconstrained nonlinear optimisation algorithm in the family of quasi-Newton methods. In general, LBFGS has better performance than other available algorithms for small datasets and provides efficient weight optimisation^1^. Each pattern was standardised before being input into the ANN by subtracting the mean and dividing by the standard deviation of the pattern.

#### 2.1.2 Modelling linear and nonlinear relationships with the ANN

Equation 1 defines a general mapping between *X* and *Y* as follows:


(1) Y=f(X)+E

where f(.) could be a linear or nonlinear function and E is a zero-mean Gaussian matrix. For nTL-MDPC, we use an ANN to have a more general estimation of f(.) than a linear regression allows. According to Equation 2, using the train subset, for any ROIs X and Y, and a set of time points $t_{x}$ and $t_{y}$ , we have:


(2) $$Y_{\text{train}}=f(X_{\text{train}})+E_{\text{train}}$$

where $X_{\text{train}}$ is the input of the ANN, which is used to predict $Y_{\text{train}}$ during training. Below is a step-by-step description of how $X_{\text{train}}$ and $Y_{\text{train}}$ are related.

Equation 3 defines the input of the *h*th node in the hidden layer:


(3) $$z_{h}=\sum_{i=1}^{N_{x}}x_{i}w_{\mathrm{ih}},h=1,\ldots ,N_{H}$$

where $x_{i}$ is the *i*th input, $w_{\mathrm{ih}}$ is the weight between the *i*th node in the input and the *h*th node in the hidden layer, $N_{x}$ is the number of nodes in the input layer, and $N_{H}$ is the number of nodes in the hidden layer.

Equation 4 obtains the output of the *h*th node in the hidden layer is as follows:


(4) $$g_{h}(z_{h})=a_{h}\tanh(b_{h}z_{h})$$

where $a_{h}$ and $b_{h}$ are scaling parameters of the tangent hyperbolic function.

Finally, the output of the *j*th node in the output layer is obtained by Equation 5 as:


(5) $$y_{j}=\sum_{h=1}^{N_{h}}g_{h}(z_{h})v_{\mathrm{hj}}=\sum_{h=1}^{N_{h}}a_{h}\tanh(b_{h}\sum_{i=1}^{N_{x}}x_{i}w_{\mathrm{ih}})v_{\mathrm{hj}},j=1,\ldots ,N_{y}$$

where $v_{\mathrm{hj}}$ is the weight between *h*th node in the hidden and *j*th node in the output layer, and $N_{y}$ is the number of nodes in the output layer.

After estimating the above parameters and function f(.) , the predicted patterns in ROI Y can be obtained by Equation 6 using the test subset as follows:


(6) $${\hat{Y}}_{\text{test}}=f(X_{\text{test}})$$

where $\;{\hat{Y}}_{\text{test}}$ is the predicted pattern in ROI Y at time point $t_{y}$ . Then, Equation 7 computes the explained variance (EV) for each vertex k = *1*,…, $n_{y}$ :


(7) $$\mathrm{EV}(Y_{\text{test}}^{k},{\hat{Y}}_{\text{test}}^{k})=1-\frac{\mathrm{var}(Y_{\text{test}}^{k}-{\hat{Y}}_{\text{test}}^{k})}{\mathrm{var}(Y_{\text{test}}^{k})}\;$$

Finally, to quantify the multidimensional connectivity metric for each pair of ROIs at each pair of time points, we summarise the above EVs using Equation 8 by averaging across all of the vertices in ROI Y:


(8) $$\mathrm{EV}(Y_{t\mathrm{est}},{\hat{Y}}_{\text{test}})=\frac{\sum_{k=1}^{n_{y}}\mathrm{EV}(Y_{\text{test}}^{k},{\hat{Y}}_{\text{test}}^{k})\;}{n_{y}}\;$$

### 2.2 Simulations: comparing the performance of linear and nonlinear TL-MDPC on different relationships between patterns

**Figure 2:**
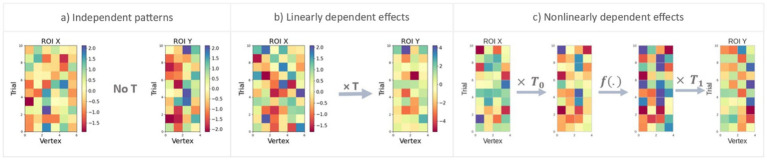
Representation of the three simulation scenarios designed to test accurate detection of different types of multidimensional connectivity. The matrices ( X and Y ) show patterns of responses in ROI X and ROI Y, respectively, with rows indicating different trials and columns indicating vertices. **(a)** In this scenario, X and Y are independent patterns with no reliable transformation between the regions, and as a result, no connectivity should be identified. **(b)** Here, there is a linear multidimensional relationship between X and Y through matrix T . **(c)** In the final scenario, nonlinear multidimensional relationships between X and Y are simulated through a neural network transformation. Patterns in ROI X are first transformed linearly through $T_{0}$ , then passed through a nonlinear (sigmoid or tangent hyperbolic) function f(.) , and the resulting patterns again transformed through $T_{1}$ to produce the patterns in ROI Y.

Before applying nTL-MDPC to EEG/MEG data, it is critical to know whether it performs as expected in simulated test scenarios where the relationships between response patterns are known, and how this performance compares to that of TL-MDPC. To this end, we created three different scenarios shown in Figure 2: (1) no relationship between the activity patterns, (2) linear multidimensional relationships, and (3) nonlinear multidimensional relationships. We assessed and compared the performance of TL-MDPC and nTL-MDPC on each scenario for reasonable and practical choices of the number of trials, vertices, and signal-to-noise ratios (SNR). Thus, we were able to test the ability of each method to detect linear and nonlinear relationships when present, and correctly identify no connections where such relationships were absent. Critically, we expected that nTL-MDPC would capture more nonlinear connectivity than TL-MDPC, while it was not clear whether it would achieve the same level of performance as the linear TL-MDPC at detecting linear connections. Note that, as the estimation of transformations is done time sample by time sample and does not rely on the precise time points of X and Y , we here assess the performance of our method without simulating time courses. Thus, we use the shorter terms MDPC and nMDPC to refer to the two methods in our simulations.

#### 2.2.1 Simulation parameters

To more thoroughly assess the performance in each scenario, we varied the critical parameters, namely the number of trials, vertices, and signal-to-noise ratios (SNRs), within realistic ranges. The number of vertices used was either 5 or 15, i.e., the minimum and maximum number of vertices obtained from the implementation of our clustering approach on our EEG/MEG dataset (Rahimi et al., 2023). To report the final connectivity values and assess the stability of connectivity estimates, each simulation condition was repeated 100 times with different random trial subsamples. Trials were randomly selected without replacement to simulate increasing data availability, and results were averaged across iterations. Standard deviations across runs were used to visualise variability, helping assess the robustness of the connectivity estimates.

#### 2.2.2 Scenario 1: checking for spurious connectivity between two independent patterns

To confirm that any apparent advantages of TL-MDPC or nTL-MDPC were not due to an overall increase in their likelihood to identify connections, we assessed whether either showed a propensity to detect false positives. Figure 2a shows this first scenario, representing two independent patterns with no relationship, so that there is no *f*(.), i.e., Y≠f(X) . To do so, two pattern matrices with random noise were generated independently using normal distributions (mean = 0 and std. = 1) across 30, 50, 100, 150, and 300 trials.

#### 2.2.3 Scenario 2: comparing the methods’ abilities to capture linear dependencies between two patterns

We then compared TL-MDPC and nTL-MDPC on their ability to detect linear multidimensional relationships over 50, 150, and 300 trials. Figure 2b shows patterns with this relationship, with vertices in ROI X uncorrelated to each other but transformed to ROI Y through a matrix *T*, so that Y=XT+E , where T is of size $n_{x}\times n_{y}$ , and E is the error term. To create these, we produced patterns *X* through a normal distribution (mean = 0, and std. = 1). For the transformation matrix, we generated matrices using a normal distribution (mean = 0, and std. = 1) with different degrees of sparsity (varying from 10% of the matrix size to 100%, with 10% as the step size). Y was computed by multiplying X and T . Different levels of noise were then introduced from a zero-mean normal distribution with a varying standard deviation (std = 10^std_pow^, where std_pow ∈ [−2, −1.5, −1, −0.5, 0, 0.5, 1, 1.5, 2]).

#### 2.2.4 Scenario 3: testing the methods’ abilities to capture nonlinear dependencies between two patterns

Scenario 3 assessed how well the two methods can identify nonlinear multidimensional relationships, and whether the performance of the nonlinear method was superior. Constructing nonlinear relationships is not as straightforward as making the patterns for the two previous scenarios, as there are infinitely many ways to simulate a nonlinear function. Here, we chose a method that is easily tractable yet inspired by our knowledge of neuronal interactions. We mimicked the process which determines neuronal activation and firing patterns, a linear summation of weighted inputs followed by nonlinear scaling, to create activation patterns that could reflect the kinds of nonlinear relationships found in the brain.

As Figure 2c illustrates, the patterns in ROI X were first linearly transformed through $T_{0}$ and the resulting outputs passed through a nonlinear function. We utilised both sigmoid and tangent hyperbolic functions as these are considered similar to how neurons summarise information (Sharma et al., 2017). The resulting matrix then passes through another transformation matrix, $T_{1}$ , yielding *Y*. Specifically, we first generated X and $T_{0}$ using a normal distribution (mean = 0, and std. = 1). The output of the first layer is gained through multiplication of X with $T_{0}$ ( $Y_{0}=XT_{0}$ ). Second, this (linear) output is fed through a nonlinear sigmoid or tangent hyperbolic function ( $Y_{1}=f(Y_{0}),\text{where}\;y_{\mathrm{ij}}^{1}=(\frac{1}{1+e^{-y_{\mathrm{ij}}^{o}}}),\text{or}\;y_{\mathrm{ij}}^{1}=\tanh(y_{\mathrm{ij}}^{0})$ ). The resulting patterns are then transformed through $T_{1}$ to produce patterns in ROI Y, so that $Y=Y_{1}T_{1}+E,$ where E is a zero-mean Gaussian noise of size $n_{t}\times n_{y}$ . We introduced zero-mean noise from a normal distribution with a variable standard deviation (std = 10^std_pow^, where std_pow ∈ [−2, −1.5, −1, −0.5, 0, 0.5, 1, 1.5, 2]). As in scenario 2, both $T_{0}$ and $T_{1}$ were generated with different degrees of sparsity, and the same number of trials, vertices, and replications was used.

### 2.3 Comparing nTL-MDPC to TL-MDPC in a real EEG/MEG dataset

We then applied nTL-MDPC to an existing EEG/MEG dataset (for more details, see Farahibozorg, 2018; Rahimi et al., 2022) to determine whether it would identify additional connections between nodes of the semantic network due to its improved ability to detect nonlinear multidimensional connections. This dataset allows a comparison of deeper (in the semantic decision task; SD) over shallower (in the lexical decision task; LD) semantic processing and is therefore expected to show large differences in connectivity within the semantic network. The connectivity of this dataset has been assessed previously with linear TL-MDPC, allowing direct comparison (Rahimi et al., 2023).

#### 2.3.1 Participants

Our EEG/MEG dataset contains recordings from 18 healthy native English speakers (mean age 27.00 ± 5.13, 12 female) with normal or corrected-to-normal vision. The experiment was approved by the Cambridge Psychology Research Ethics Committee, and volunteers were paid for their time and effort.

#### 2.3.2 Stimuli and procedure

The experiment included 250 words and 250 pseudowords. It comprised four blocks presented in a random sequence. One of the four blocks used a lexical decision (LD) task and the other three a semantic decision (SD) task. In the LD block, participants were asked to decide if the presented stimulus was referring to a word or a pseudoword. In SD blocks, they were required to decide whether the presented word was referring to a certain category of words, namely “non-citrus fruits,” “something edible with a distinctive odour,” and “food containing milk, flour or egg.” A stimuli of 10% belonged to these target categories and required a button-press response. As in our previous studies, we only analysed brain responses to real, non-target words. Each stimulus was presented for 150 ms, with an average Stimulus Onset Asynchrony of 2,400 ms. A summary of the stimuli is presented in Supplementary Figure S1.

#### 2.3.3 Data acquisition and preprocessing

MEG/EEG recordings were collected simultaneously using a Neuromag Vectorview system (Elekta AB, Stockholm, Sweden) and MEG-compatible EEG cap (EasyCap GmbH, Herrsching, Germany) at the MRC Cognition and Brain Sciences Unit, University of Cambridge, United Kingdom. MEG was acquired via a 306-channel system consisting of 204 planar gradiometers and 102 magnetometers. EEG was collected via a 70-electrode system with an extended 10–10% electrode layout. The data sampling rate was 1,000 Hz.

As we intended to directly compare the nTL-MDPC results to those previously published using TL-MDPC, the preprocessing and source estimation steps are identical to Rahimi et al. (2023). We used MEGIN Maxwell-Filter software to apply signal source separation (SSS) with its spatiotemporal extension to remove noise from spatially distant sources and to compensate for small head movements (Taulu and Kajola, 2005). We used the MNE-Python software package (Gramfort et al., 2013, 2014) to perform the preprocessing and source reconstruction. The raw data from each participant was visually checked, and bad EEG channels were selected for interpolation (max = 9 channels per person, min = 0, mean = 2.85). A finite-impulse-response (FIR) bandpass filter between 0.1 and 45 Hz was applied, as well as the FastICA algorithm to remove eye movement artefacts (Hyvarinen, 1999; Hyvärinen and Oja, 2000). Afterwards, epochs were created using the data from 300 ms pre-stimulus to 600 ms post-stimulus. A flowchart of the different steps of pre-processing is shown in Supplementary Figure S2.

#### 2.3.4 Source estimation

L2-Minimum Norm Estimation (MNE) (Hämäläinen and Ilmoniemi, 1994; Hauk, 2004) was used for source estimation. We assembled inverse operators based on a 3-layer Boundary Element Model (BEM) of the head geometry, gained from structural MRI images. For this purpose, we assumed sources to be perpendicular to the cortical surface (“fixed” orientation constraint). We obtained the noise covariance matrices using 300 ms-baseline periods and then selected the best choice from a group of methods included in MNE-Python (‘shrunk’, ‘diagonal_fixed’, ‘empirical’, and ‘factor_analysis’) (Engemann and Gramfort, 2015). MNE-Python’s default SNR = 3.0 was used to regularise the inverse operator for evoked responses. The source signals from each participant were then morphed to the standard Freesurfer brain (fsaverage).

#### 2.3.5 Regions of interest

As in Rahimi et al. (2023), we used six regions of interest including left and right anterior temporal lobe (ATL), left inferior frontal gyrus (IFG), left posterior temporal cortex (PTC), left angular gyrus (AG) and left primary visual area (PVA) to study connectivity within the semantic network. These ROIs were constructed using the Human Connectome Project (HCP) parcellation (Glasser et al., 2016). An analysis of leakage among these ROIs can be found in our previous paper (Rahimi et al., 2023). To assess and quantify potential spatial leakage among our six ROIs, we previously computed a leakage matrix based on point spread functions (PSFs) derived from the resolution matrix of our source estimates (Rahimi et al., 2022). This matrix indicated the degree to which signal from one ROI leaked into others, compared to self-leakage. The results showed that all ROI pairs exhibited low to medium leakage, except for slightly higher leakage from lATL to PTC. Importantly, this pattern did not correspond to the strongest observed connectivity, suggesting that our multidimensional connectivity results are unlikely to be driven primarily by leakage artefacts.

#### 2.3.6 Application of nTL-MDPC to real EEG/MEG data

We performed nTL-MDPC to predict X from Y and Y from X at every 25 ms from 100 ms pre-stimulus to 500 ms post-stimulus and reported the EVs (averaged across the two directions) as the final metric. EVs for every pair of ROIs and across latencies are presented in TTMs (Rahimi et al., 2023). In any TTM, each row indicates statistical relationships between Y at a specific time point and X at each time point, while columns show statistical relationships between X at a certain time point and Y across all time points, enabling us to display connectivity at different time lags. A flowchart illustrating the connectivity estimation process is presented in Supplementary Figure S3.

To avoid any potential bias due to the different number of trials between SD and LD tasks (Bastos and Schoffelen, 2016), TTMs were computed independently for each of the three SD blocks and then averaged, allowing a better comparison with LD. SD and LD connectivity were then contrasted using non-parametric cluster-based permutation tests, implemented in MNE Python (Maris and Oostenveld, 2007). This approach was chosen because it does not assume normality, making it suitable for high-dimensional EEG/MEG data, and it provides a built-in correction for multiple comparisons (Candia-Rivera and Valenza, 2022) by evaluating the significance of clusters as a whole, rather than at individual time-lag coordinates. We implemented two-sided t-tests thresholded at a t-value corresponding to an alpha-level of 0.05 and 5,000 randomised repetitions. To avoid spurious or small clusters, we only presented clusters whose size exceeded 2% of the TTMs total size, i.e., with more than 12 elements.

The TTM results derived from both the TL-MDPC and nTL-MDPC methods were analysed to determine whether there was a significant difference in the effects captured by each approach. Specifically, we aimed to assess whether the nonlinear nTL-MDPC approach demonstrated greater connectivity—either in terms of magnitude or across additional lags—compared to the TL-MDPC method. This comparison was conducted using cluster-based permutation tests with the same parameters and statistical configurations as in the previous paragraph, including two-sided t-tests thresholded at a t-value corresponding to an alpha-level of 0.05 with 5,000 randomised repetitions. Notably, this comparison was performed at the cluster level rather than the individual cell level, focusing exclusively on clusters with sizes greater than 12 elements (i.e., clusters larger than 2% of the TTM’s total elements) to avoid spurious or small clusters. Through these tests, we evaluated whether the nonlinear adjustments in the nTL-MDPC method revealed any additional or distinct significant effects between the SD and LD conditions that were not evident when using the linear TL-MDPC method.

## 3 Results

### 3.1 Simulation results

#### 3.1.1 Scenario 1: independent patterns

**Figure 3:**
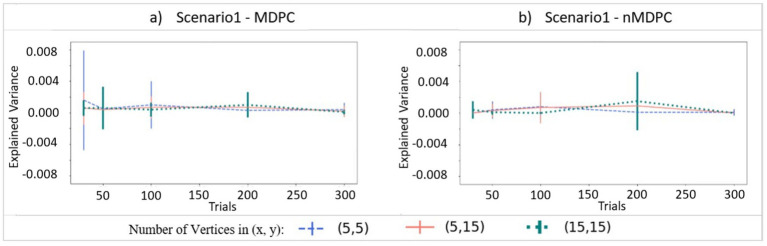
Connectivity values (explained variance, y-axis) for two independent patterns. **(a)** Connectivity for (linear) MDPC as a function of different numbers of trials, with different curves representing different combinations of numbers of vertices in ROI X and ROI Y. **(b)** Similar to **(a)**, but for nMDPC. The values are close to zero indicating that neither MDPC nor nMDPC methods are prone to false positive errors for random patterns. Note that while all EVs and their means were positive (as negative EVs were replaced by zeros), error bars based on standard deviations can still extend below zero.

Figure 3 demonstrates connectivity scores (EV, y-axis) for the case where there is no true relationship between two patterns, with the x-axis showing different numbers of trials and different curves representing different numbers of vertices for both panels. For both MDPC (panel a) and nMDPC (panel b), all values are close to zero with highly overlapping error bars, suggesting that neither method is prone to false positive errors when no relationship exists. The fluctuations in explained variance reflect expected random variability across runs due to trial-to-trial differences, the stochastic nature of the training–testing framework, and the overall small magnitude of EV. These variations do not indicate an inherent sensitivity of MDPC or nMDPC to trial numbers but rather reflect noise across repeated simulations.

#### 3.1.2 Scenario 2: linear multidimensional dependency between two patterns

**Figure 4:**
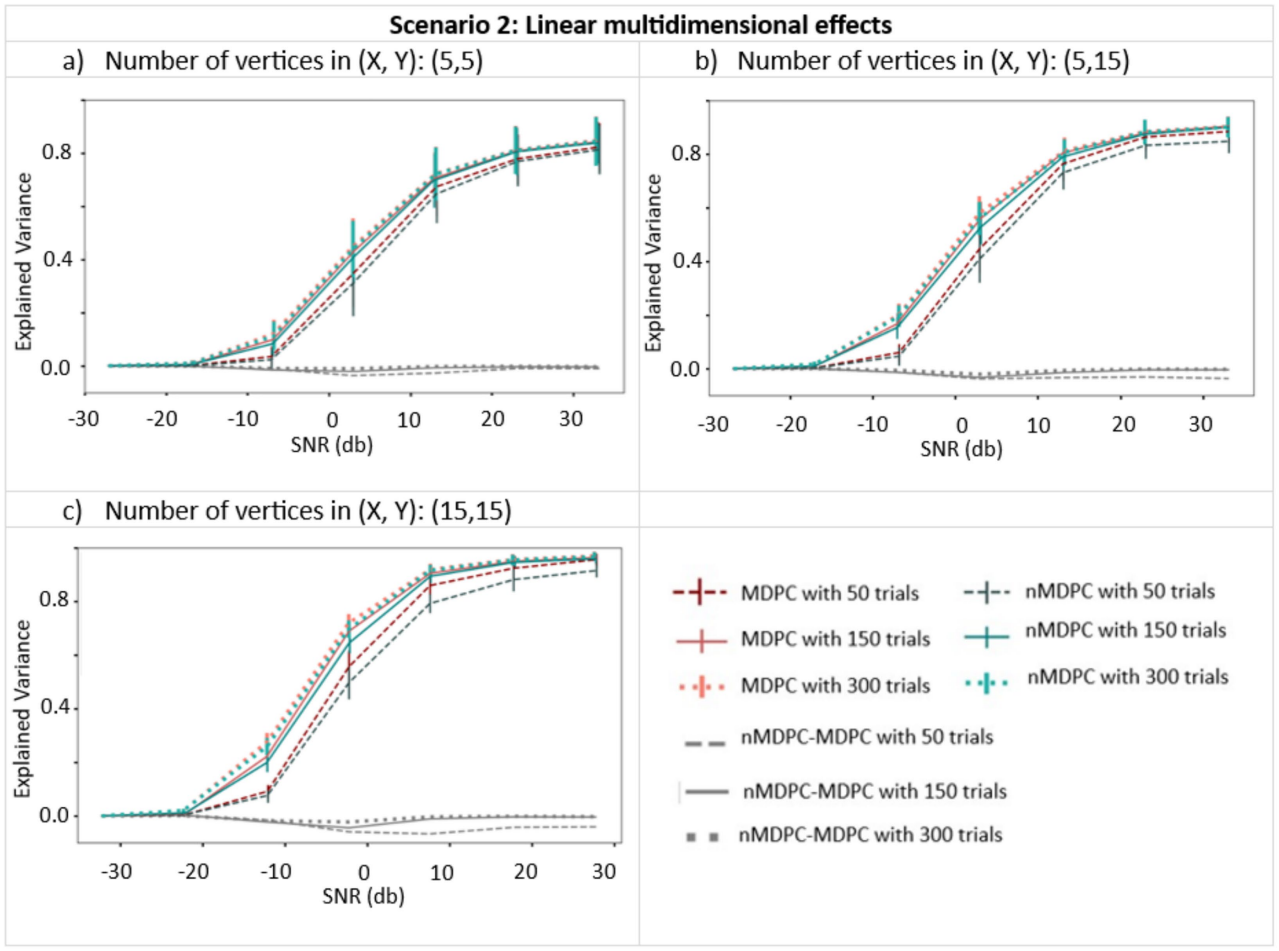
Connectivity metrics for linear multidimensional effects assessed using MDPC and nMDPC. Connectivity metrics for linear multidimensional effects assessed using MDPC and nMDPC for three different numbers of vertices: **(a)** (X, Y) = (5, 5), **(b)** (X, Y) = (5, 15), and **(c)** (X, Y) = (15, 15). All panels show connectivity scores (explained variance, y-axis) between patterns that have a true linear multidimensional dependency, for different SNRs (x-axis), numbers of trials (MDPC approach: red curves, nMDPC approach: blue curves), and number of vertices (three panels). All cases show that EV reaches values above 0.8 for SNRs above 25 db, and is close to zero for SNRs smaller than −10 db. In all panels, both linear and nonlinear MDPC show similar results, with MDPC producing greater EV.

Figure 4 demonstrates how well the MDPC and nMDPC methods perform in the case where multidimensional patterns are linearly related. Red curves show the result of the MDPC method, and blue curves represent the nMDPC results. For both methods and in all cases, EV reaches values between 0.8 and 1 for SNRs above 25 db and is close to zero for SNRs smaller than −10 db. For intermediate SNRs, the two methods perform similarly with highly overlapping error bars, with EV rising for increasing numbers of trials. Thus, both linear and nonlinear MDPC similarly capture true linear relationships between patterns to a similar degree, with MDPC producing slightly greater EV values.

#### 3.1.3 Scenario 3: nonlinear multidimensional dependency between two patterns

**Figure 5:**
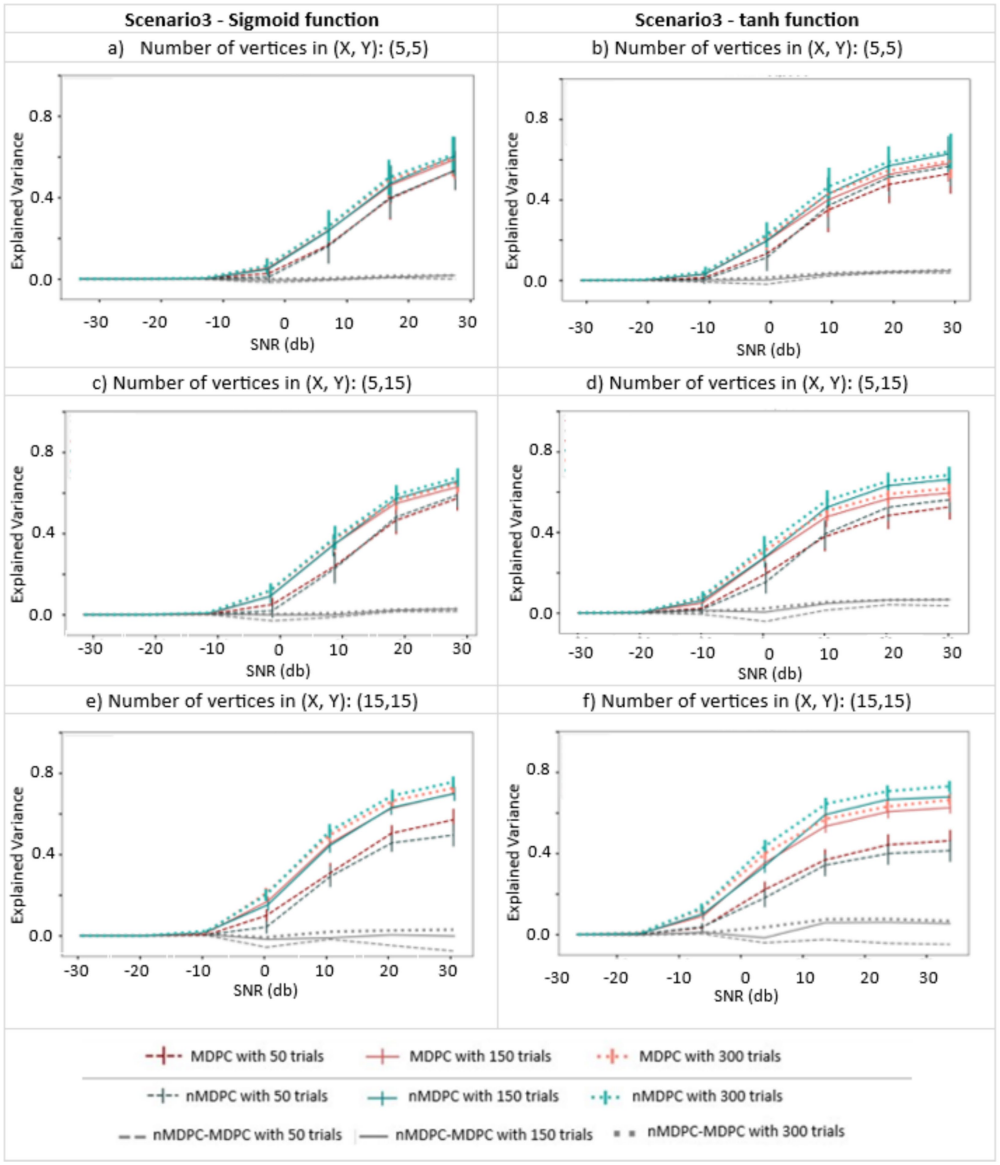
Connectivity metrics for nonlinear multidimensional effects using MDPC and nMDPC. **(a,c,e)** Connectivity scores (explained variance, y-axis) between patterns with nonlinear multidimensional dependency, for different SNRs (x-axis), three different numbers of trials (MDPC approach: red curves, nMDPC approach: green curves) and different number of vertices (three panels), using sigmoid as the activation function. **(b,d,f)** Same as **(a,c,e)** but with a tangent hyperbolic activation function. Linear MDPC provides a good approximation to the nonlinear scenarios in most cases, and even performs slightly better than nMDPC for low number of trials and large number of vertices. However, nMDPC generally explains some additional variance, particularly for larger numbers of trials (e.g., a trials-to-vertex ratio ≥ 10:1).

Figure 5 shows the connectivity (EV, y-axis) between pairs of multidimensional patterns with a nonlinear relationship, for different SNRs (x-axis), trials (indicated by line type), and numbers of vertices (three panels), generated using sigmoid (left panels) and tangent hyperbolic (right panels) activation functions. EV is around zero for all cases at very low SNRs (<−10 dB). Unlike for linear dependencies, the EV does not approach 1 at high SNRs. Nonlinear MDPC generally outperforms its linear counterpart, with a numerical difference of approximately 0.1 and with little overlap of error bars. Figures 5e,f show that the worst performance for both methods is obtained with the largest number of vertices and the lowest number of trials. Interestingly, in this case, the linear method outperforms the nonlinear one. This is likely due to the known requirement of ANNs for a lot of training data (Hagan and Demuth, 1999). Thus, linear MDPC provides a good approximation of the nonlinear multidimensional relationships, particularly when a large number of parameters needs to be estimated from a small number of trials. Yet, in general, nMDPC captures some more variance. This improved ability of nMDPC to detect additional nonlinear relationships could allow the identification of additional functional connections in brain data, including connectivity between more regions or across additional time lags.

### 3.2 Comparison of nTL-MDPC and TL-MDPC in a real EEG/MEG dataset

**Figure 6:**
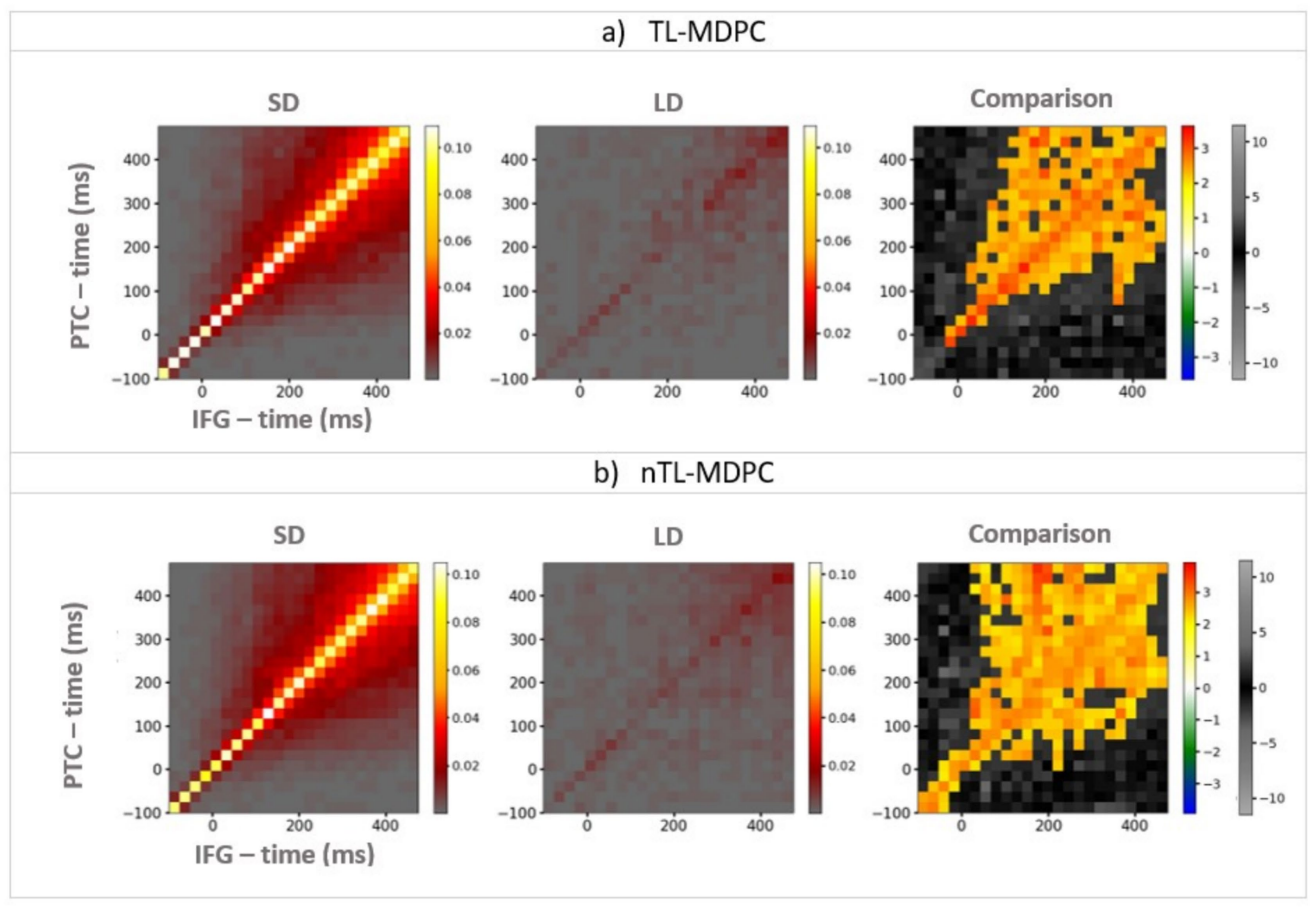
An example of TTMs showing the connectivity between PTC (y-axis) and IFG (x-axis), for the semantic decision (SD) task (the left column), the lexical decision (LD) task (the middle column), and their comparison (i.e., connectivity that is greater when there are greater semantic demands; right column). **(a)** TTMs for TL-MDPC, **(b)** TTMs for nTL-MDPC. Colour bars show connectivity scores (explained variance) for the first two left columns. For the third column, the hot and cold colour bar highlights significant effects obtained from the cluster-based permutation test, whereas the grey-scale colour bar shows non-significant t-values (this colour bar is the same across all figures). With both methods, the greatest connectivity occurs around the diagonal; however, the statistical comparison between the tasks reveals more reliable modulation of connectivity in the nTL-MDPC case, particularly at later latencies and time lags. Note that this does not necessarily reflect a significant difference between the connectivity identified using the two methods (see below).

As nTL-MDPC outperformed TL-MDPC in detecting nonlinear relationships in our simulations, we went on to test whether this is also the case for real EEG/MEG data previously analysed with TL-MDPC, which contrasts a semantic decision with a lexical decision task. We asked whether nTL-MDPC detects additional modulations in dynamic connectivity when the need for deep semantic processing is greater, compared to TL-MDPC. Figure 6 shows the connections identified in each task using either TL-MDPC (Figure 6a) or nTL-MDPC (Figure 6b) for one pair of ROIs (the PTC and IFG) to illustrate how the task difference results are obtained. The TTM is computed separately for the SD and LD tasks, which are then statistically compared (using cluster-based permutation tests) to generate the final TTM. An element of the TTM at coordinate (*x,y*) describes the relationship between patterns in IFG at latency *x* and PTC at latency *y*. Note that while a significant cluster in TTMs indicates a significant statistical relationship between patterns in two regions across two latencies, it does not formally demonstrate directionality of the corresponding connectivity, even at non-zero time lags. Relationship across time lags may be assumed to be due to the influence of the area at the earlier time point on the area at the later time point. However, this influence may not be direct and cannot be assumed to be causal (see Discussion section). As expected, both approaches identified greater connectivity for the more semantically demanding SD task compared to the LD task. The TTMs for SD and LD have a similar shape, with the largest values concentrated around the diagonal. However, the statistical comparison between the tasks highlights the more reliable identification of connectivity for nTL-MDPC around the diagonal, as well as across larger time lags later in the trials.

It is important to note that low explained variance (EV) does not necessarily indicate a complete lack of connectivity. Instead, it may arise due to patterns unseen during training, noise, or insufficient data. This is a challenge faced by all connectivity methods. For instance, spectral connectivity methods can fail to detect meaningful relationships when applied to the wrong frequency range. Similarly, Granger causality methods require stationarity within a given time window, potentially missing transient connectivity patterns. Ultimately, different connectivity methods capture different aspects of brain dynamics, and no single approach can account for all variations.

For all other ROI pairs, the TTMs for each individual task are presented in Supplemental Figure S4.

#### 3.2.1 Detecting connectivity within the semantic network across time with nTL-MDPC

**Figure 7:**
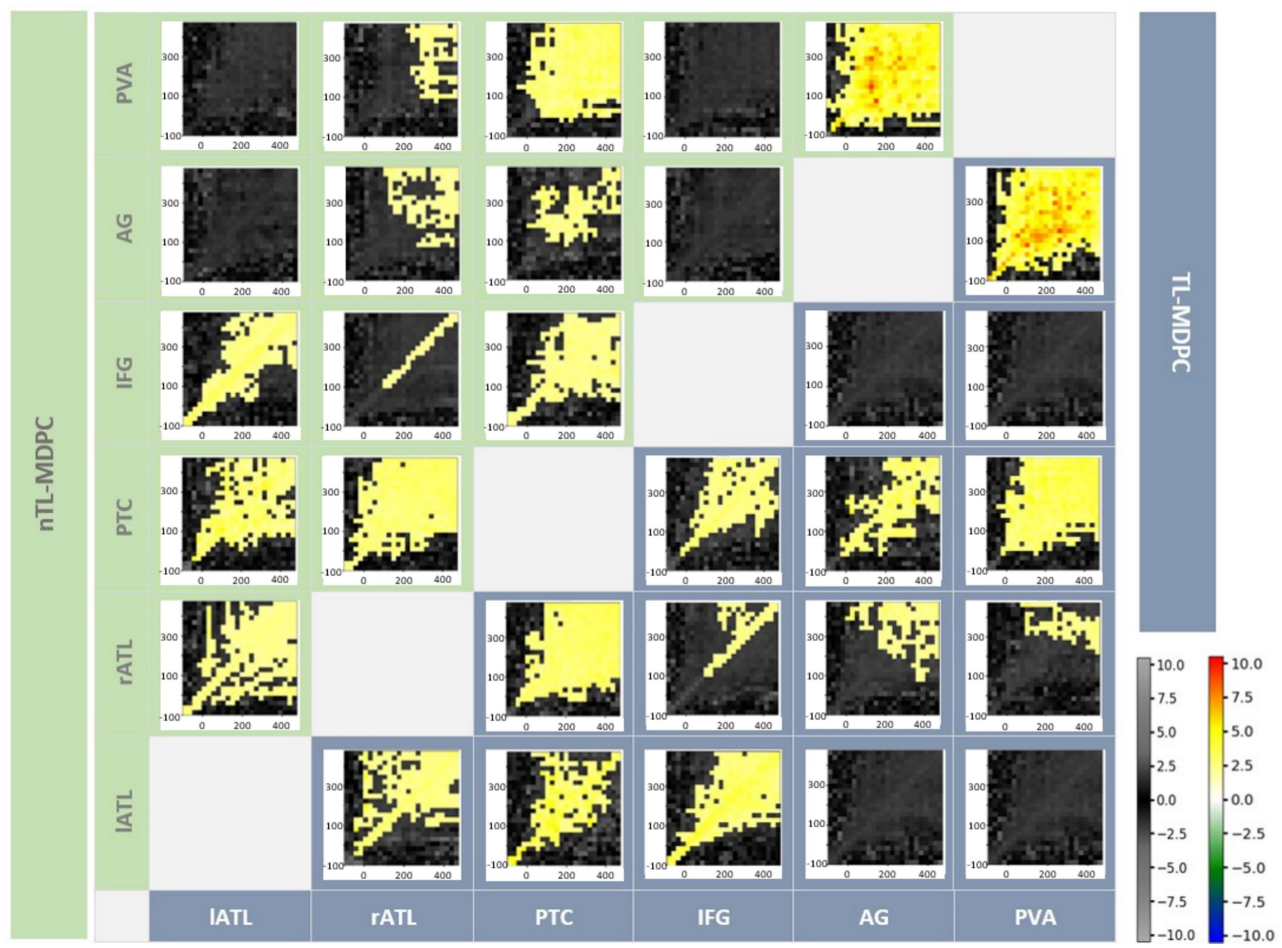
Inter-regional Connectivity Matrix (ICM) for semantic network modulations in the brain—the upper diagonal (green shaded area) shows nTL-MDPC TTMs and the lower diagonal (blue shaded area) shows TL-MDPC TTMs. Each TTM reflects EVs for a pair of regions, ROI X and ROI Y, and across time. All modulations with both methods showed greater connectivity for SD than LD. Cluster size was thresholded at 2% of TTM’s size (24*24). Using both methods, we found rich modulations between semantic control and representation regions. The hot and cold colour bar highlights significant effects obtained from the cluster-based permutation test, whereas the grey-scale colour bar shows non-significant t-values. The colour bars are the same across all figures. There were no significant differences between the results for the two methods.

We computed the functional connectivity of all possible pairs of the six semantic ROIs using nTL-MDPC. Figure 7 shows the inter-regional connectivity matrix (ICM) (Rahimi et al., 2023), a summary of the semantic decision versus lexical decision TTMs for all ROI comparisons, with the upper diagonal (green area) showing the nTL-MDPC results, and the lower diagonal (blue area) displaying the TL-MDPC results. Generally, nTL-MDPC identified greater connectivity with deeper semantic processing between the same pairs of regions as TL-MDPC (in lATL-rATL, lATL-PTC, lATL-IFG, rATL-PTC, rATL-IFG, rATL-AG, rATL-PVA, PTC-IFG, PTC-AG, PTC-PVA, and AG-PVA). As found with TL-MDPC, the connectivity between core semantic regions (including lATL-rATL, lATL-PTC, lATL-IFG, rATL-PTC, and PTC-IFG), as well as AG-PVA, appears to “fan out” from the diagonal over time, i.e., connectivity persists over longer time lags at later latencies on both sides of the diagonal, possibly reflecting bidirectional or recurrent information flow (McClelland and Rumelhart, 1989; Rogers and McClelland, 2014; Clarke et al., 2018; Kietzmann et al., 2019). Connectivity was also identified over short time lags for rATL-IFG, and late effects were revealed for rATL-AG and rATL-PVA.

#### 3.2.2 Direct comparison of nTL-MDPC with TL-MDPC

The results of nTL-MDPC were overall similar to those of TL-MDPC, and indeed, no additional ROI pairs were found to be connected. However, nTL-MDPC captured more connectivity in some cases. Specifically, for some pairs, it revealed significant modulations across a greater number of time lags, particularly those over greater time windows, e.g., between lATL-rATL, lATL-PTC, rATL-PTC, and PTC-IFG. However, these differences were subtle, and care should be taken when interpreting cluster size with cluster-based thresholding. Moreover, when we formally contrasted the two methods directly using cluster-based permutation tests, there were no significant differences at the cluster level (note that cell-wise comparisons were not conducted).

#### 3.2.3 Visualising the variation in early and late connections in the semantic network across connectivity methods

**Figure 8:**
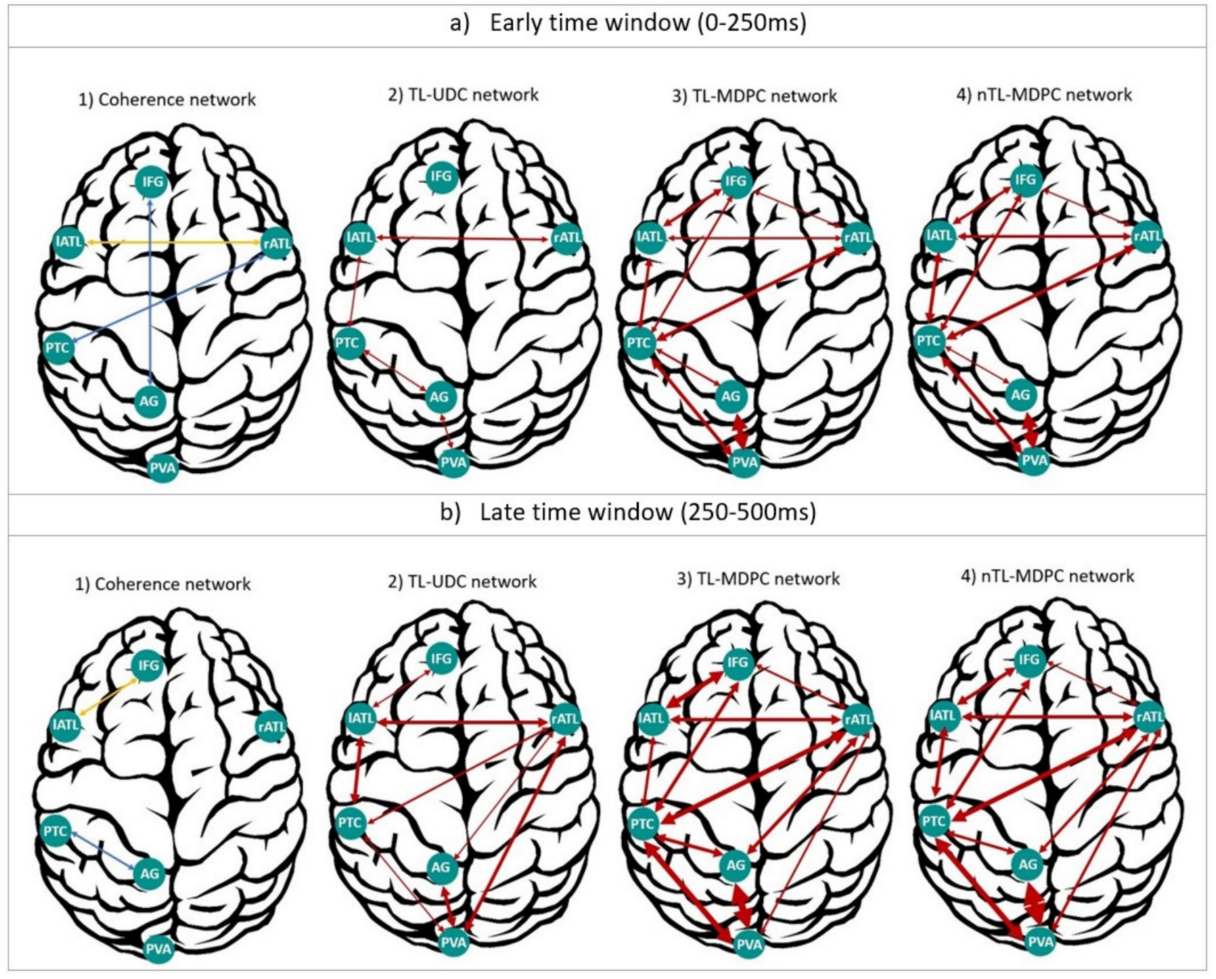
Summary of our cortical functional connectivity analyses of EEG/MEG data using four different connectivity approaches. **(a)** Networks extracted for an early time window (0–250 ms), through (1) coherence (Rahimi et al., 2022), (2) the UDC method (Rahimi et al., 2023), (3) MDPC (Rahimi et al., 2023), and (4) nMDPC. **(b)** Same as **(a)**, but for a late time window (250–500 ms). For coherence, the blue connections represent dependencies specific to the gamma band, and yellow ones show the connections consistent across the alpha, beta, and gamma bands. For the other three methods, arrows represent the summed significant t-values in each time window as a metric of connection intensity, with thicker arrows reflecting more intense connections (higher summed t-values) and vice versa. Overall, TL-MDPC reveals more connections compared to the two unidimensional methods, but TL-MDPC and nTL-MDPC produced the same pattern of connectivity with very few differences.

As multiple different methods have now been used to assess connectivity within the same dataset, we summarised the networks extracted using these different methods in Figure 8. This demonstrates the results from: (1) coherence in four frequency bands (Rahimi et al., 2022), (2) TL-UDC (Rahimi et al., 2023), (3) TL-MDPC (Rahimi et al., 2023), and (4) nTL-MDPC analyses, for early (50–250 ms) and late (250–500 ms) time windows. TL-UDC is the unidimensional counterpart of TL-MDPC (Rahimi et al., 2023), in which all the time courses of the vertices within each ROI are collapsed into one, allowing a direct assessment of the effects of multidimensionality. As coherence was computed per frequency band, we represent the connections specific to the gamma band in blue (i.e., rATL-PTC, IFG-AG at the first time window, and PTC-AG at the second time window), and connections consistent across three frequency bands (i.e., alpha, beta, and gamma) in yellow. To summarise across time lags for TL-UDC, TL-MDPC, and nTL-MDPC, we summed the significant t-values in each time window as a metric of connection intensity. This reflects increases in connectivity strength and time lags with significant connectivity across longer time lags, indicating stronger or more reliable connectivity across time and participants. We represented these values with the arrow widths (with the weakest connectivity reflected by the thinnest lines, as in rATL-IFG with nTL-MDPC at the earlier time window, and the strongest connectivity represented by the thickest line, as in AG-PVA with nTL-MDPC at the later time window). Note that, unlike coherence-based connectivity metrics—which assess phase synchronisation between signals, typically within narrow frequency bands—TL-MDPC and nTL-MDPC estimate the extent to which the multidimensional pattern of activation in one ROI at time t_1_ can predict the pattern in another ROI at time t_2_. These methods quantify connectivity as the proportion of explained variance (EV) in the target pattern, based on a trained transformation function (e.g., an artificial neural network in the nonlinear case). As such, they capture statistical dependencies—both linear and nonlinear-across multivariate spatiotemporal activation patterns, rather than oscillatory coupling. This distinction is critical for understanding what aspects of brain dynamics each method operationalises.

In general, the two unidimensional connectivity methods revealed fewer and weaker task modulations of connectivity, whereas both multidimensional methods detected richer connectivity across the whole semantic network across the whole latency range. Both multidimensional methods identify rich interactions between the four core semantic representation and control regions (lATL, rATL, PTC, and IFG) starting at early time points (around the onset of the stimulus) and becoming stronger across the time window. Even with methods designed to identify nonlinear relationships, the connectivity of AG is relatively sparse. It only shows connectivity with PVA and PTC from earlier time points, and with rATL at later latencies. The general pattern of results was similar for TL-MDPC and nTL-MDPC, with both distinguishing a core semantic network comprising lATL, rATL, PTC, and IFG from a posterior visual attention network consisting of AG, PVA, and PTC, with overlap of these two sub-networks in PTC.

While the contrast between our linear and nonlinear TL-MDPC TTMs did not yield any significant effects, visual inspection of Figure 8 reveals some subtle numerical differences between the two approaches. Using nTL-MDPC, we found that the connectivity values were numerically higher for lATL-rATL and PTC-IFG at early time points, and for lATL-PTC, PTC-IFG, and rATL-PVA at the later time points. However, connectivity values were numerically higher for lATL-IFG and PTC-AG when using the linear TL-MDPC method at the late time window. The finding of a greater number of numerically stronger connections for the nonlinear method could suggest some subtle benefits for this method in the overall reliability of the evidence of connectivity between the regions of the semantic network for the two methods. However, these differences are very limited, and they did not result in any significant differences in the statistical comparison between the TTMs.

## 4 Discussion

We introduced a novel multidimensional functional connectivity method, nTL-MDPC, to capture the nonlinear dependencies between event-related EEG/MEG activation patterns across different brain regions and at different time lags. Tests using simulated data revealed that both nTL-MDPC and TL-MDPC can capture linear and nonlinear multidimensional relationships, and neither is prone to false positive errors for independent and uncorrelated patterns. For true nonlinear dependencies, nTL-MDPC generally outperforms the linear method, except when low number of trials is combined with high number of vertices. However, the linear method also captured a substantial amount of variance, indicating that nonlinear relationships can often be approximated well using the linear method. This was confirmed in the real EEG/MEG data analysis, where the linear and nonlinear methods showed a highly similar performance with no significant differences. We combined EEG and MEG for source estimation to optimise spatial resolution and have previously demonstrated that this pattern of results was unlikely to be caused by leakage. The results of our new nonlinear approach were generally in line with our previous findings with TL-MDPC, indicating that linear TL-MDPC may provide a good and efficient approximation to nonlinear multidimensional relationships between brain regions in real EEG/MEG data. Indeed, reviewing the results across multiple connectivity methods demonstrates a far greater need for multidimensional rather than nonlinear methods. Given the differential computational and time demands, linear multidimensional methods may be a convenient and efficient choice for future functional connectivity assessments of EEG/MEG.

This study is a key step towards characterising the full dynamic multidimensional information in vertex-to-vertex relationships across brain regions. Our main findings are that (1) in the simulations, both methods could identify some linear and nonlinear relationships, with the nonlinear method performing slightly better, and (2) in our real data analysis, we did not observe significant differences between the two methods. Thus, we cannot report a significant advantage of using a computationally more demanding nonlinear over a simpler linear method, in contrast to the fMRI analyses by Anzellotti et al. (2017a, 2017b). While nTL-MDPC theoretically should capture additional nonlinear relationships, the practical findings indicate that TL-MDPC remains an effective and computationally efficient alternative. There are two possible explanations for these results. First, there may be no nonlinear relationships in the EEG/MEG data, or second, there may be nonlinear relationships in this data and our linear method may be able to approximate them well enough—rather than the nonlinear method failing to detect them—within the noise level. Our simulations suggest that the latter scenario is likely, as linear multidimensional methods can approximate nonlinear dependencies well. This takeaway is an important conclusion of our study, highlighting that linear multidimensional methods may often suffice for EEG/MEG functional connectivity analyses. Thus, TL-MDPC may perform better under violations of the assumption of linearity than expected, further supporting the use of this method. It is not yet clear why this differs from the prior fMRI findings. It seems unlikely that the difficulty is the lack of nonlinear connections in this specific dataset, based on the need for nonlinear transformations in semantic tasks. It may be that the improved temporal resolution of EEG/MEG data allowed the linear method to estimate nonlinear relationships better, a finding that should be confirmed in other datasets. Alternatively, the signal-to-noise ratio of EEG/MEG data may be insufficient to identify nonlinear relationships. However, there is likely to be variation across datasets. The relative performance of linear and nonlinear methods would be expected to depend on the SNR in the dataset, the number of trials and dimensions utilised, and the number of participants, as demonstrated by the simulations. Thus, future studies should investigate whether the ability of TL-MDPC to identify both linear and nonlinear relationships is a general property of EEG/MEG data or whether there are cases for which the nonlinear method is more suitable (for instance, when attempting to identify subtle connectivity changes across many trials).

The performance of TL-MDPC may also depend on the type of nonlinearity present. Here, we focused on testing particular nonlinear functions that we expect to be likely to exist within the brain, yet there are an infinite number of possible nonlinear relationships. Testing all of these would clearly be impossible, and any attempt to do so would be beyond the current single study. It is also possible to decompose nonlinear relationships into linear and nonlinear parts, as for example shown for multivariate connectivity methods (Talebi et al., 2019). This would allow the estimation of linear and nonlinear connectivity components separately within a dataset, allowing greater interpretation of the kinds of connections present in EEG/MEG data and informing future methodological advances. Of course, we cannot discount that there may also be additional nonlinear connections within the data that we are currently not identifying with either method.

The ANN used in nTL-MDPC captures statistical dependencies between multidimensional patterns using a feedforward mapping that includes a nonlinear activation function, e.g., tanh. While the complexity of these models makes direct interpretation of the learned transformations unlikely- and they are often treated as black-box methods- the explained variance metric still offers a useful quantitative index of how well nonlinear dependencies are captured. By combining the method with simulations, we can evaluate the method’s sensitivity to different types of underlying relationships, helping to establish its ability to detect both linear and nonlinear interactions. Although interpreting the precise nature of these mappings in the context of semantic processing remains a valuable goal, it is beyond the scope of the current study, which primarily focuses on comparing the performance of linear and nonlinear connectivity methods in capturing meaningful dependencies; however, the observed increases in explained variance-especially under high trial counts-suggest that the method may be able to uncover pattern relationships that could be missed by linear models. Future work may explore approaches to decompose these mappings into interpretable components or visualise the contribution of individual vertices.

As there are many possible ways to perform functional connectivity analyses, and relaxing any methodological assumptions increases the resource requirement and interpretation complexity, we may never be able to take all factors into consideration. We therefore need to know the most effective aspects. Our findings suggest that moving from unidimensional to multidimensional information reveals a more expansive network of connections, adding a great deal to our understanding of its functional interactions, yet converting a linear method to a nonlinear version does not. Combined with the easily interpretable transformation matrix and reduced computational demands of the linear method, this may mean the linear TL-MDPC method is a reasonable and pragmatic choice for future connectivity research. Thus, the current research is expected to help focus our aims on the important aspects for future methods development and applications.

Both TL-MDPC (Rahimi et al., 2023) and nTL-MDPC allowed more detailed investigation of the semantic network across space and time when applied to dynamic EEG/MEG data, compared to previous studies on this network using fMRI (Jackson et al., 2016; Jung and Lambon Ralph, 2016; Chiou et al., 2018) and unidimensional analyses with EEG/MEG (Sormaz et al., 2017; Rahimi et al., 2022). Both identified two sub-networks connected through PTC, namely a semantic network comprised of four semantic representations and control regions (left and right ATL, PTC, and IFG), and a visual-attention network consisting of PVA and AG. These results are in concordance with the controlled semantic cognition (CSC) framework, which emphasises the need for a bilateral semantic hub (ATLs) as well as the interaction between representation and control regions for the task-relevant processing of conceptual information (Lambon Ralph et al., 2016). Indeed, across our unidimensional and multidimensional analyses, there was early activation and connectivity between the ATLs. However, the multivariate methods also demonstrated how semantic task demands modulate their rich connectivity with the rest of the semantic network, including rATL connectivity with IFG and PTC in the left hemisphere. This cross-hemisphere connectivity is unlikely to be due to leakage, instead likely reflecting increased engagement of rATL for more semantically demanding tasks (Jung et al., 2019; Rahimi et al., 2022; Stefaniak et al., 2022). In contrast, another putative hub region, AG, only showed relatively sparse connectivity with posterior brain areas. This supports the findings of Farahibozorg et al. (2022), who used Dynamic Causal Modelling to study word concreteness effects and identified ATL as a connectivity hub in an early (0–250 ms) and AG only in a prolonged (0–450 ms) time interval. These results suggest a role of AG, for example, in context integration, episodic memory, or attentional processes (Chambers et al., 2004; Wagner et al., 2005; Cabeza et al., 2008, 2012; Vilberg and Rugg, 2008; Shimamura, 2011; Noonan et al., 2012, submitted manuscript^2^; Humphreys and Lambon Ralph, 2015; Humphreys et al., 2021).

In summary, linear and nonlinear multidimensional methods appear similar in their ability to detect functional connections within EEG/MEG data. While multidimensional methods are crucial for revealing novel insights into the dynamics of brain networks, linear methods may be sufficient in many cases.

That said, our study included only healthy participants. Future work involving clinical populations may uncover differential contributions of nonlinear effects across groups. Moreover, as the current sample size may have limited our ability to detect subtle nonlinear patterns, applying nTL-MDPC to large-scale datasets could offer deeper insights into when and how nonlinear relationships emerge in functional connectivity.

Knowing how to best direct our efforts may improve our chances of opening new avenues for a richer understanding of dynamic brain connectivity. While the current methods allow us to estimate statistical relationships between regions across time lags, they are not formally directional and therefore do not establish directionality or causality. Relationships across time lags may be assumed to reflect the influence of one region on another. However, this influence may not be direct—it could result from indirect connectivity (e.g., via a third region). Indeed, temporal precedence does not necessarily imply causal influence, particularly within nonlinear systems (Arinyo-i-Prats et al., 2024).

In the future, this limitation could be addressed by extending our methods to a multivariate and multidimensional approach, considering full activity patterns across multiple brain regions simultaneously. Such multivariate autoregressive models could be combined with the principles of Granger causality to establish directional and potentially causal functional connectivity between multidimensional response patterns (Seth, 2010).

## Data Availability

The data analysed in this study is subject to the following licenses/restrictions: data are available upon request. Requests to access these datasets should be directed to olaf.hauk@mrc-cbu.cam.ac.uk.

## Footnotes

^1^ https://scikit-learn.org/stable/modules/generated/sklearn.neural_network.MLPRegressor.html

^2^Noonan, K. A., Jefferies, E., Visser, M., and Lambon Ralph, M. A. (2012). Aligning evidence from functional neuroimaging and neuropsychology for the neural network underpinning semantic control: a meta-analytic investigation. (submitted manuscript)
