# Supplementary material for: Linear and nonlinear multidimensional functional connectivity methods reveal similar networks for semantic processing in EEG/MEG data
Source: Front Hum Neurosci. 2025 Jul 30;19:1533034. doi: 10.3389/fnhum.2025.1533034 (PMC12343681; doi:10.3389/fnhum.2025.1533034)
Supplement: Supplementary file 1 [file Data_Sheet_1.docx]

**Supplementary Material**


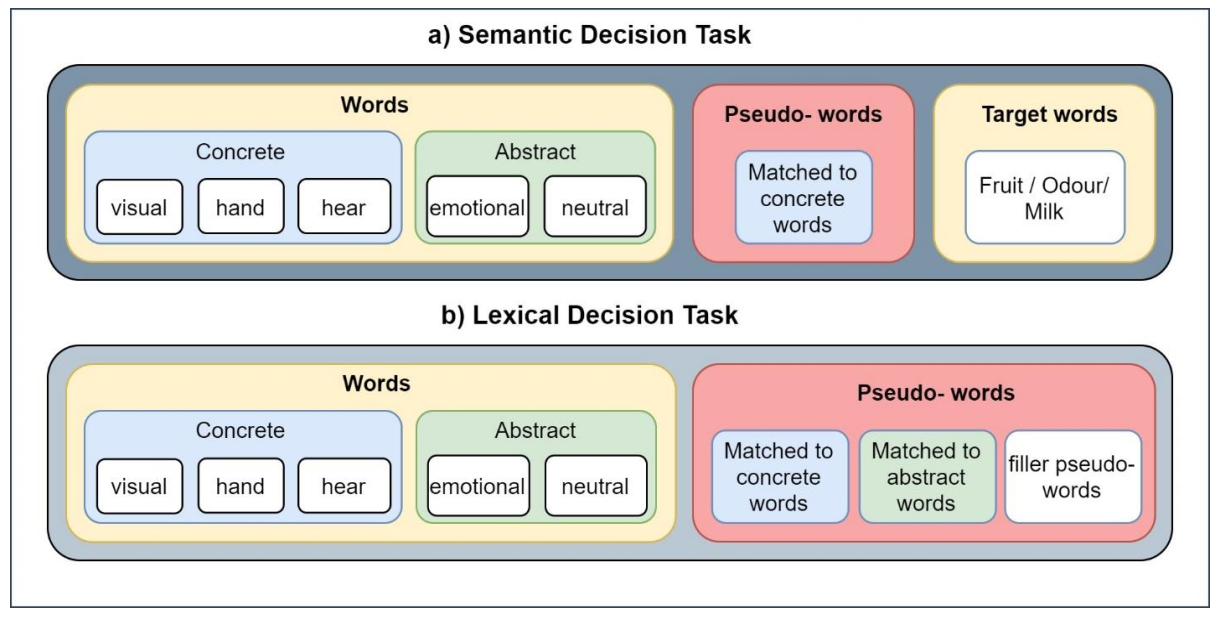


Figure S1- Schematic illustration of the experimental paradigm. a) In three blocks of the semantic task, 280 words and 50 pseudo words presented to participants, including 3 subcategories of concrete, 2 subcategories of abstract, and 1 group of pseudo words, each with 50 items, and 30 targets. Participants were asked to quietly read the strings of letters as they appeared on the screen and make button press responses with the middle finger of their left hand whenever a target appeared on the screen. Targets of the blocks were selected from three groups of “non-citrus fruits”, “something edible with distinctive odour” and “food that contains milk, flour or egg”. b) Participants were asked to carry out a lexical decision task with 250 words and 250 pseudo words, and to make button press responses with the index and ring fingers of their left hand indicating whether the following string of letters refers to a meaningful word. Blocks and stimuli were presented in a random order.


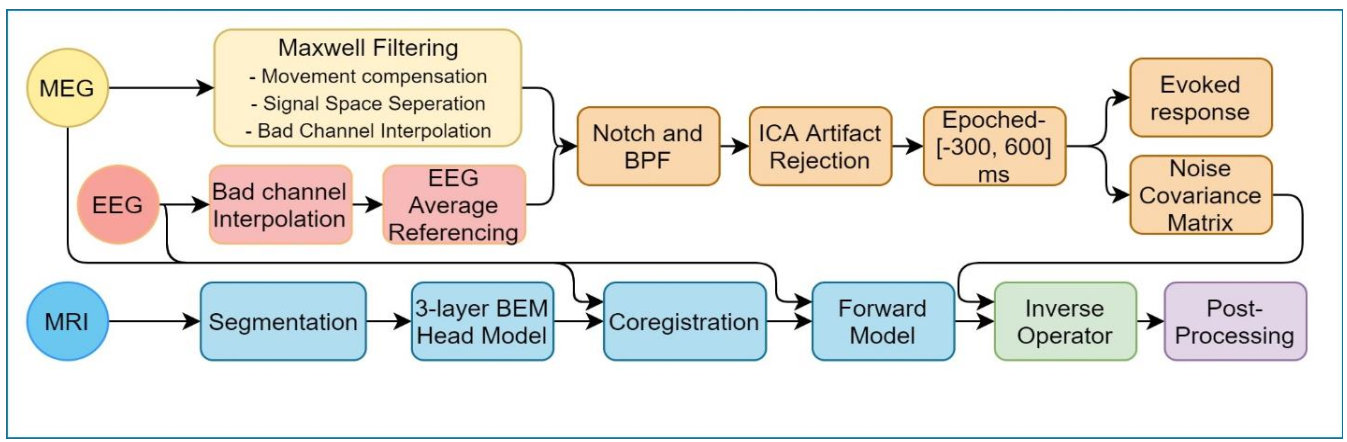


Figure S2- A flowchart of different steps of pre-processing


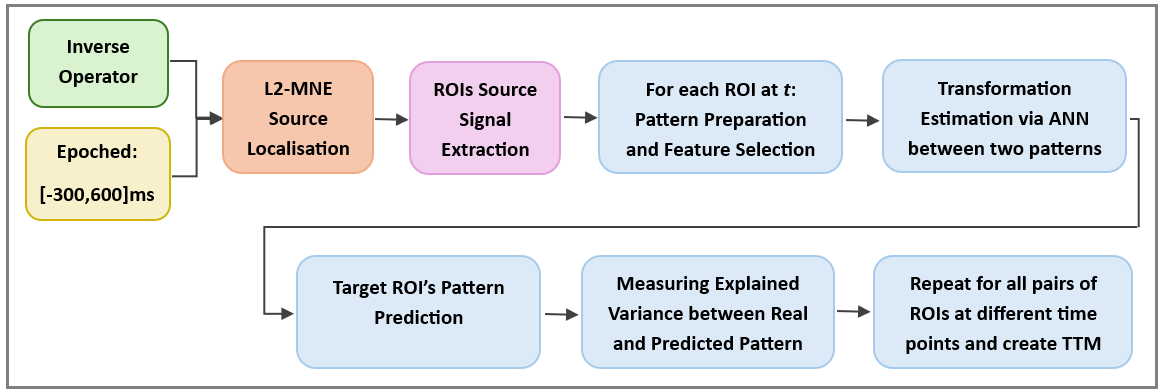


Figure S3- A flowchart of the post-processing steps employed

|  | **TL-MDPC** | **nTL-MDPC** |
| --- | --- | --- |
|  | \| **SD** \| **LD** \| **Comparison** \| \| --- \| --- \| --- \| | \| **SD** \| **LD** \| **Comparison** \| \| --- \| --- \| --- \| |
| **lATL-rATL** | 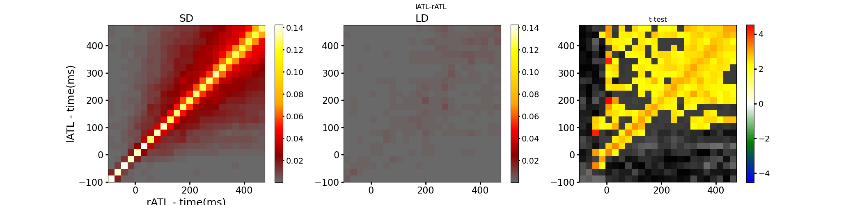 | 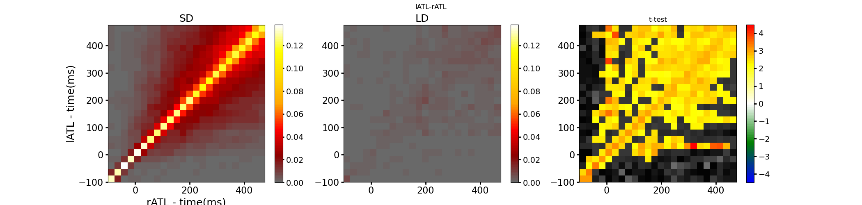 |
| **lATL-PTC** | 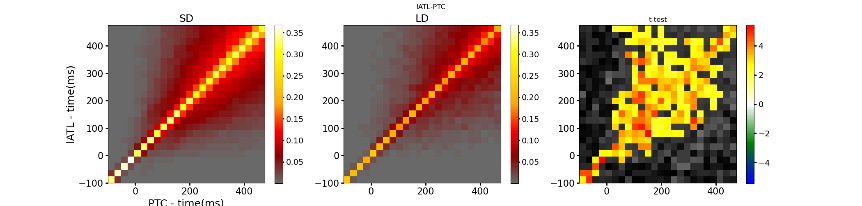 | 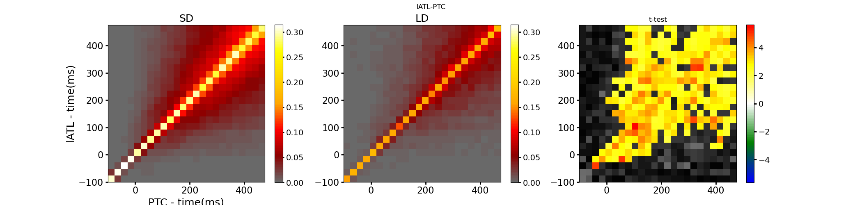 |
| **lATL-IFG** | 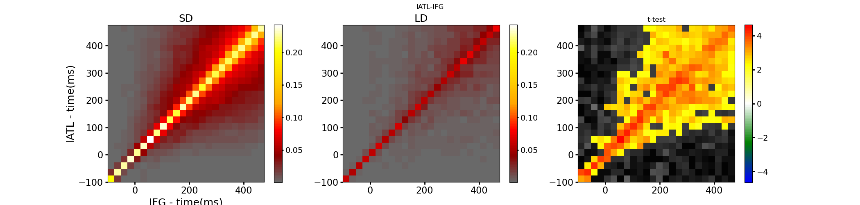 | 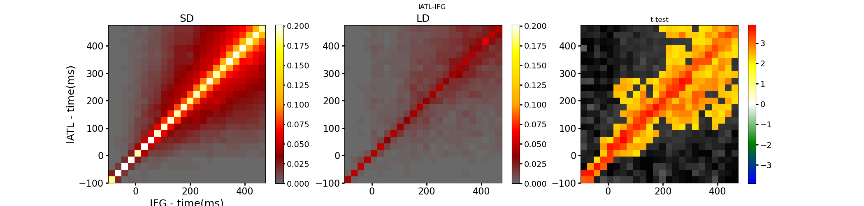 |
| **lATL-AG** | 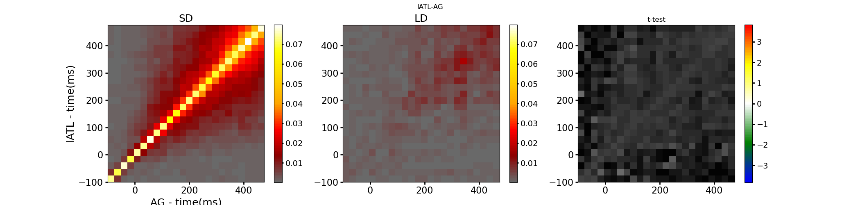 | 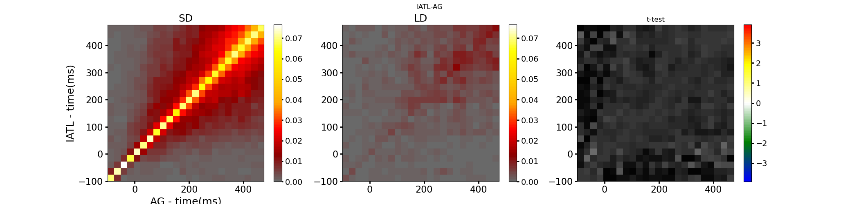 |
| **lATL-PVA** | 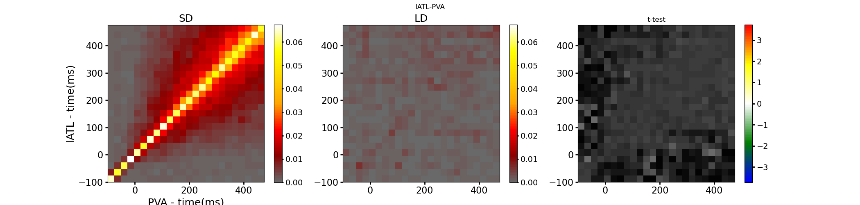 | 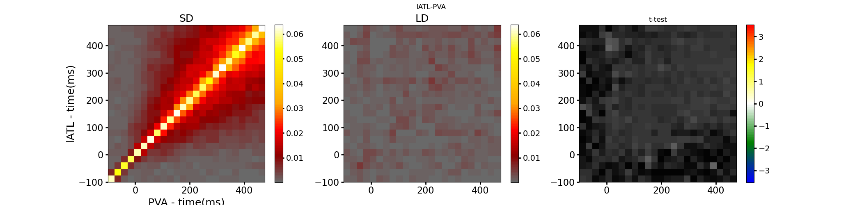 |
| **rATL-PTC** | 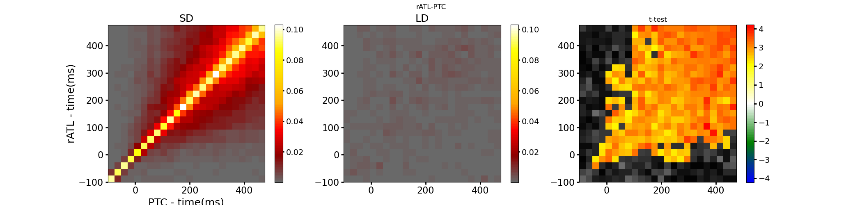 | 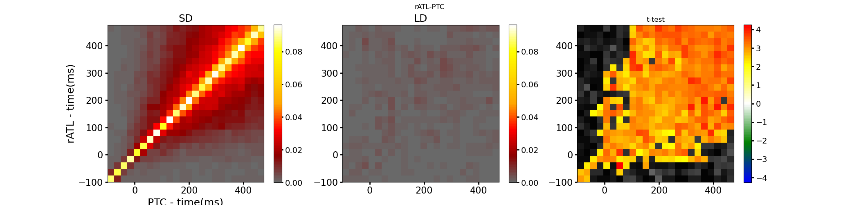 |
| **rATL-IFG** | 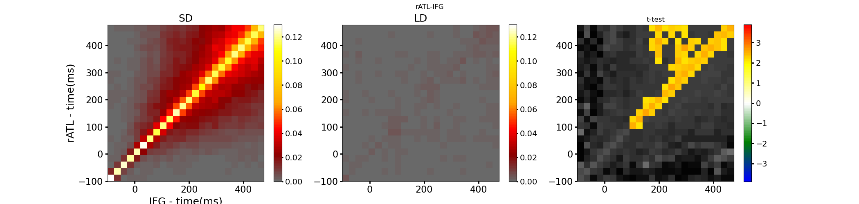 | 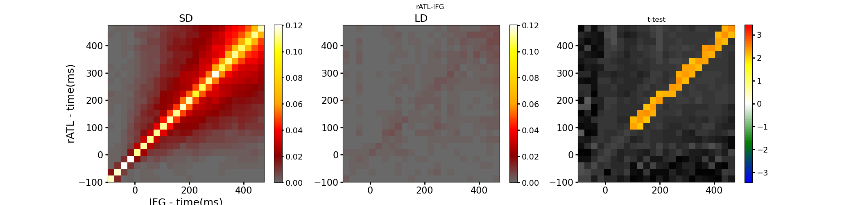 |
| **rATL-AG** | 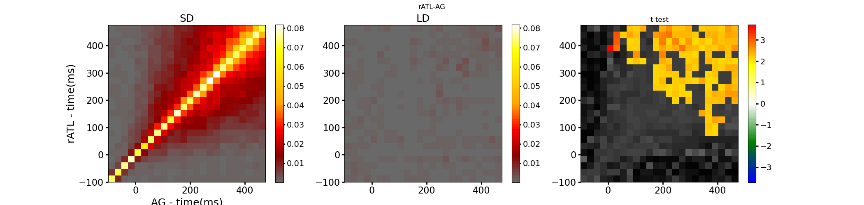 | 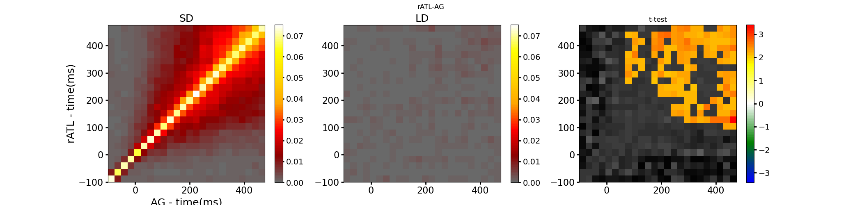 |
| **rATL-PVA** | 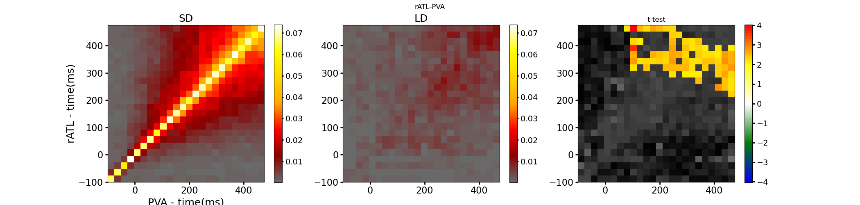 | 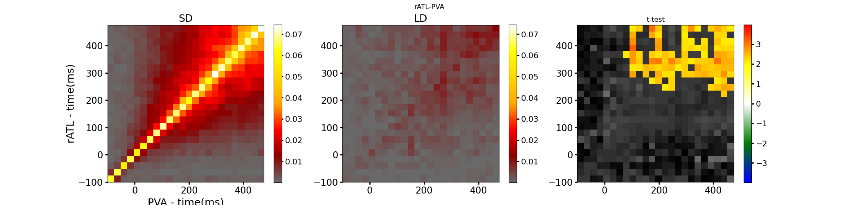 |
| **PTC-IFG** | 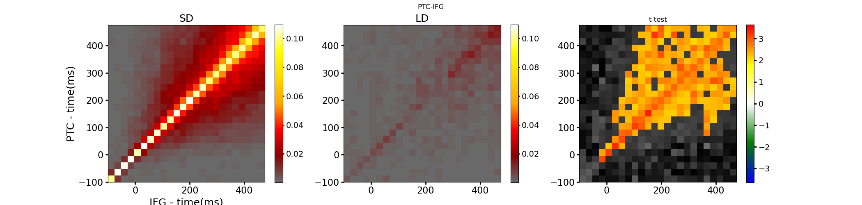 | 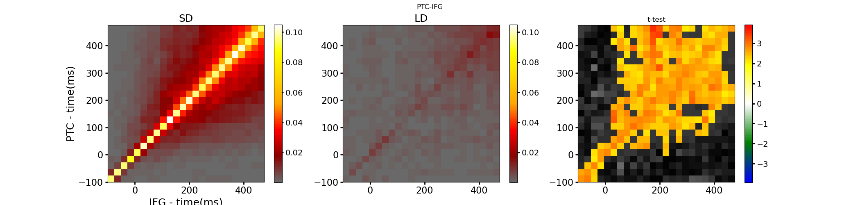 |
| **PTC-AG** | 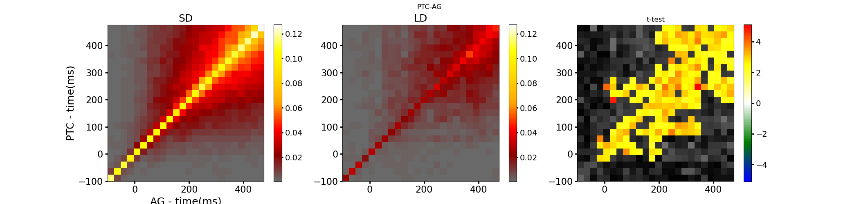 | 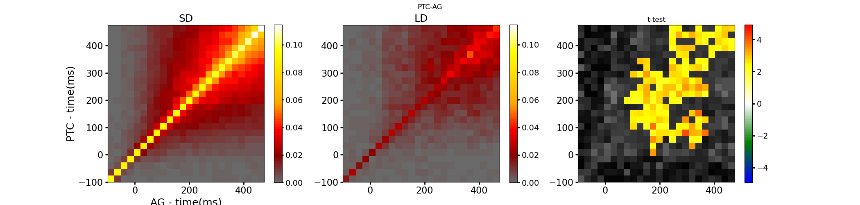 |
| **PTC-PVA** | 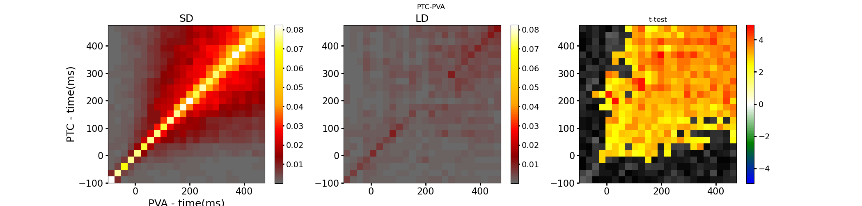 | 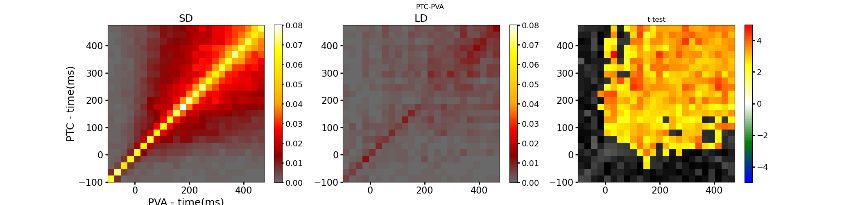 |
| **IFG-AG** | 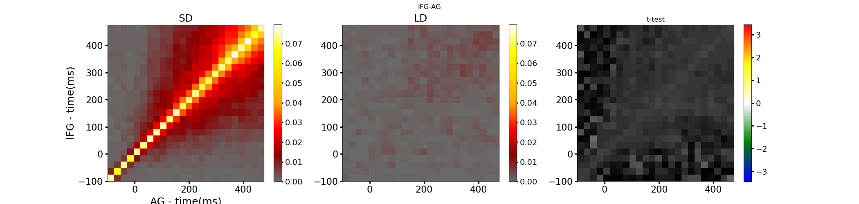 | 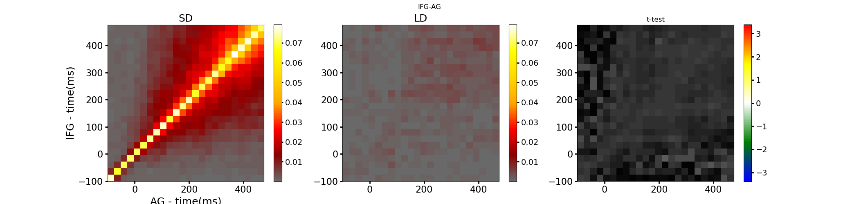 |
| **IFG-PVA** | 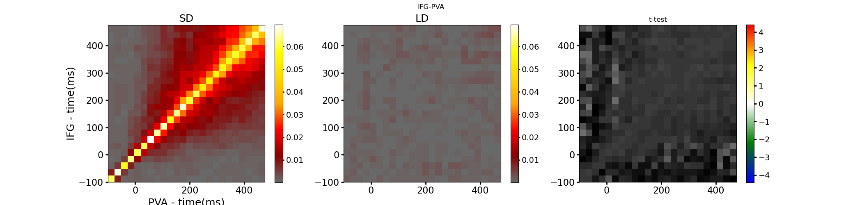 | 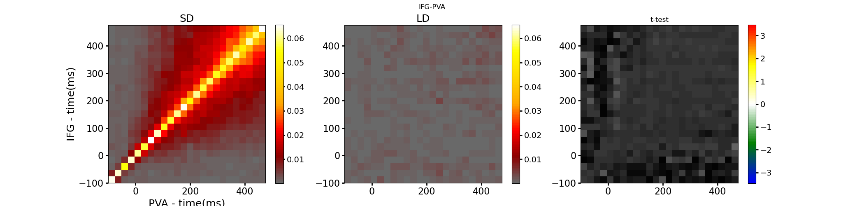 |
| **AG-PVA** | 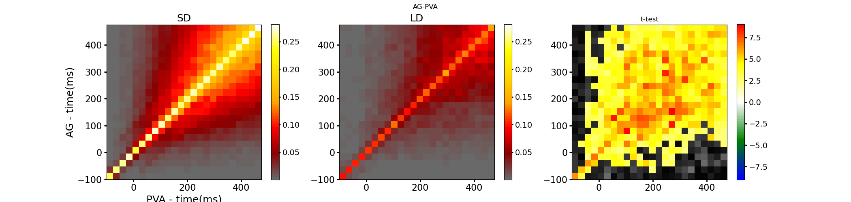 | 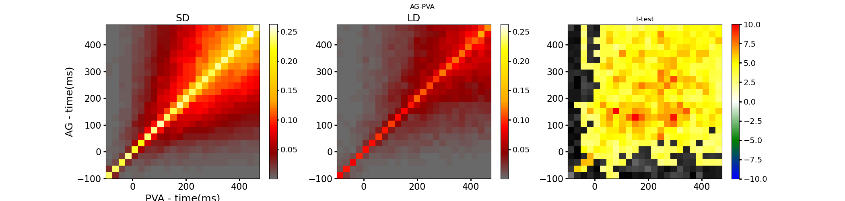 |

Figure S4- Representation of all TTMs for semantic decision (SD) and lexical decision (LD) tasks, and their comparison, using TL-MDPC (left column) and nTL-MDPC (right column). TTMs for SD and LD are averaged TTMs across participants, and comparison was done using cluster-based permutation test with alpha-level=0.05. All significant contrasts show greater connectivity for SD using MD.
